# Which level is responsible for gluteal pain in lumbar disc hernia?

**DOI:** 10.1186/s12891-016-1204-7

**Published:** 2016-08-22

**Authors:** Guofang Fang, Jianhe Zhou, Yutan Liu, Hongxun Sang, Xiangyang Xu, Zihai Ding

**Affiliations:** 1 Anatomical Institute of Minimally Invasive Surgery, Southern Medical University, Guangzhou, 510515 China; 2 Department of Orthopedics, Shenzhen Hospital of Southern Medical University, NO.1333, Xinhu Road, Shenzhen, 518100 China; 3 Spine Department of Kanghua Hospital, Dongguang city, Guangdong Province China; 4 Department of Anesthesia, Shenzhen Hospital of Southern Medical University, NO.1333, Xinhu Road, Shenzhen, 518100 China

**Keywords:** LBP, Gluteal pain, LDH, Percutaneous endoscopic lumbar discectomy

## Abstract

**Background:**

There are many different reasons why patients could be experiencing pain in the gluteal area. Previous studies have shown an association between radicular low back pain (LBP) and gluteal pain (GP). Studies locating the specific level responsible for gluteal pain in lumbar disc hernias have rarely been reported.

**Methods:**

All patients with lumbar disc herniation (LDH) in the Kanghua hospital from 2010 to 2014 were recruited. All patients underwent a lumbar spine MRI to clarify their LDH diagnosis, and patients were allocated to a GP group and a non-GP group. To determine the cause and effect relationship between LDH and GP, all of the patients were subjected to percutaneous endoscopic lumbar discectomy (PELD).

**Results:**

A total of 286 cases were included according to the inclusive criteria, with 168 cases in the GP group and 118 cases in the non-GP group. Of these, in the GP group, 159 cases involved the L4/5 level and 9 cases involved the L5/S1 level, while in the non-GP group, 43 cases involved the L4/5 level and 48 cases involved the L5/S1 level. PELD was performed in both groups. Gluteal pain gradually disappeared after surgery in all of the patients. Gluteal pain recrudesced in a patient with recurrent disc herniation (L4/5).

**Conclusions:**

As a clinical finding, gluteal pain is related to low lumbar disc hernia. The L4/5 level is the main level responsible for gluteal pain in lumbar disc hernia. No patients with gluteal pain exhibited involvement at the L3/4 level.

**Electronic supplementary material:**

The online version of this article (doi:10.1186/s12891-016-1204-7) contains supplementary material, which is available to authorized users.

## Background

Among people suffer from low back pain (LBP) during their life time [1], for 80 %, their regular activity was limited due to LDH [2]. Paraspinal region pain is the most commonly encountered pain in lumbar disc hernia. Gluteal pain is also a complaint among some patients with LDH and is sometimes the only complaint. Approximately three-quarters of patients with unilateral radicular pain presented with gluteal pain [3].

However, in clinical practice, complaints of gluteal and radicular pain in the affected leg have always been diagnosed as deep gluteal syndrome (DGS). DGS is a condition in which the sciatic nerve is compressed by any structures in the deep gluteal space. The chief symptoms of DGS are buttock pain and affected leg pain. The system is similar to nerve root pain associated with LDH.4 Therefore, it is important to make a definite diagnosis for correct treatment.

Previous studies have shown an association between radicular LBP and GP [3–7]. Studies locating the specific level responsible for gluteal pain in lumbar disc hernias have rarely been reported.

## Methods

### Design

All cases of LDH in the Kanghua hospital from 2010 to 2014 were recruited. An investigator who was blinded to LDH-related gluteal pain took the patients’ histories and performed the physical examinations. Patients were evaluated for the presence or absence of gluteal pain and were allocated to a GP group and a non-GP group. All patients underwent a lumbar spine MRI to clarify their LDH diagnosis. To determine the cause and effect relationship between LDH and GP, percutaneous endoscopic lumbar discectomy (PELD) was performed to determine the relative presence of gluteal pain with LDH. Patients were evaluated again for the presence or absence of gluteal pain after surgery.

### Inclusion and exclusion criteria

The inclusion criteria were as follows: all LDH cases with PELD surgery to determine the relationship between gluteal pain and LDH. The cases in which gluteal pain disappeared after surgery were included. The exclusion criteria were as follows: we excluded patients with hip disease, piriformis syndrome, muscle strain, cancer, arthritis of the sacroiliac joints and multiple levels of LDH. The cases in which gluteal pain did not disappear after surgery were excluded.

### Follow-up evaluations

Patients were followed up regularly by the operating surgeon for 24 months after their surgeries. The incidences of complications in the surgery groups, including the incidence of neurological complications and ODI improvement rates, were assessed.

### Data analysis

The associations between level and gluteal pain and the incidence of neurological complications were evaluated in a 2 × 2 contingency table. ODI improvement rates were evaluated using a Mann-Whitney Test.

## Results

From 2010 to 2014, 286 cases were included according to the inclusion criteria. Table 1 provided participant characteristics at baseline. Of these, 168 cases had gluteal pain (GP group) and 91cases had no pain in the gluteal area (non-GP group). Patients in the GP group ranged in age from 18 to 68 years, with a mean age of 39.7 years. Patients in the non-GP group ranged in age from 20 to 63 years, with a mean age of 41.0 years. All of the patients presented symptoms and confirmatory signs of lumbar radiculopathy that were consistent with the symptomatic disc level and MRI imaging findings.

**Table 1:** Patient demographic characteristics

	GP group (*n* = 168)	non-GP group (*n* = 118)
Age	39.7 ± 9.6	41.0 ± 10.9
Gender (male: female)	122:46	80:38
Level		
L3/4	0	27
L4/5	159	43
L5/S1	9	48

Table 2 presented the association between GP and LDH. In the GP group, 159 cases involved the L4/5 level, accounting for 94.6 %, and 9 cases involved the L5/S1 level, accounting for 5.4 %. In the non-GP group, 43 cases involved the L4/5 level, and 48 cases involved the L5/S1 level. L4/5 level involvement accounted for 94.6 % of the GP group, larger than the involvement of the L5/S1 level (*P* < 0.001), indicating that the L4/5 level was a primary cause in gluteal-related LBP. No cases of gluteal pain involved the L3/4 level.

**Table 2:** Association between gluteal pain and lumbar disc hernia

	L3/4	L4/5	L5/S1
GP group	0	159 (78.7 %)	9 (15.8 %)
Non-GP group	27	43 (22.3 %)	48 (84.2 %)
Statistical parameter			X^2^ = 77.23
*P*-value			*P* < 0.001

Percutaneous endoscopic lumbar discectomy surgeries were performed in the GP group. In both the GP and non-GP groups, the mean ODI values decreased significantly after surgery (from 69.3 ± 8.1 to 13.5 ± 7.0, *p* < 0.001, and from 71.0 ± 7.9 to 15.1 ± 7.9, *p* < 0.001, respectively). A total of 63.1 % of the patients in the GP group felt no pain after surgery. One month after their surgeries, gluteal pain disappeared in all of the patients in the GP group, which indicated that LDH was responsible for gluteal pain. The pain appeared again in a patient with L4/5 disc recurrence, confirming the hypothesis.

With regard to the incidence of neurological complications in the GP group, there were 19 cases of lower limb numbness during surgery, accounting for 11.3 % of the GP group and including two cases of foot drop and two cases of cerebrospinal leakage. These patients recovered 6–12 months after surgery. Regarding the incidence of neurological complications in the epidural anaesthesia group, there were 11 cases of lower limb numbness during surgery, accounting for 10.2 % of the non-GP group and including one case of foot drop and two cases of cerebrospinal leakage. These patients recovered 6–12 months after surgery. No significant difference was observed regarding the incidences of neurological complications between the two groups (*P* > 0.05).

## Discussion

Gluteal pain may derive from hip disease, piriformis syndrome, muscle strain, cancer and arthritis of the sacroiliac joints [8–10]. Because the symptoms are similar, LDH-related gluteal pain can be misdiagnosed as superior cluneal nerve entrapment neuropathy (SCNEN) or muscle strain. Yasuhiro Chiba [3] reported five patients with intermittent LBP due to SCNEN who had previously received conservative treatment and who underwent surgery and achieved good results. Adelmanesh F [4, 6] reported that trigger points in the superior-lateral quadrant of the gluteal area are highly specific indicators for radicular LBP. Thiese MS [5] found that patients with LBP had pain in the gluteal areas. All of the studies reported an association between radicular LBP and gluteal pain. However, the specific level responsible for gluteal pain in lumbar disc hernia has rarely been reported.

We could not completely rule out those patients with hip disease and musculoskeletal disease only by physical examination, electromyography and MRI. To avoid mixing the results and to identify the casual relationship between GP and LDH, PELD was induced to remove prominent nucleus pulposus, and GP was then re-evaluated after surgery. The disappearance of GP after surgery meant that the GP was caused by LDH. Only the cases in which GP disappeared after surgery were included in this study.

From this study, in the GP group, 159 cases involved the L4/5 level, accounting for 94.6 %; only nine cases involved the L5/S1 level, accounting for 5.4 %. Thus, the L4/5 level was a predominant culprit in gluteal-related LBP, and the results did not indicate a correlation between the L3/4 level and gluteal-related LBP. Among cases of L4/5 level involvement with lumbar disc herniation, 78.7 % had gluteal pain, while among cases with L5/S1 level involvement with lumbar disc herniation, only 15.8 % gluteal pain. No cases of L3/4 level involvement with lumbar disc herniation had gluteal pain.

All of the cases were subjected to percutaneous endoscopic lumbar discectomy, and gluteal pain subsided gradually post-surgery in all of the cases. Gluteal pain relapsed in one patient with L4/5 disc recurrence. This finding could imply a cause and effect relationship between LDH and gluteal pain. L4/5 disc hernia was a predominant factor in LDH-related gluteal pain.

PELD has become a standard procedure in recent years as a result of several advantages. Because PELD surgery only removes the hernia nucleus pulposus and does not disrupt the other structures of the lower back, the results are not confounded. Post-operative dysesthesia (POD) due to existing dorsal root ganglion (DRG) injury is a unique complication of PELD that accounts for the majority of neurological complications. As shown in Table 3, the two groups exhibited no significant differences with regard to incidences of neurological complications and ODI improvement rates.

**Table 3:** Comparison of clinical results between the GP group and the non-GP group

	Neurological complications	ODI improvement rate
GP group	11.3 %	85.6 %
Non-GP group	10.2 %	85.8 %
Statistical parameter	X^2^ = 0.237	Z = 0.633
*P*-value	0.626	0.527

The clinical implications of this study are as follows. Firstly, because the results suggest a clear relationship between L4/5 disc herniation and gluteal pain, GP assessment could help in diagnosing patients with L4/5 disc herniation. Secondly, beyond the accepted therapies aimed at the gluteal area, PELD may be suggested if the diagnosis of LDH is clarified, which might improve outcomes. This hypothesis should be tested in further anatomical studies.

## Conclusions

This study is a retrospective study in which only the cases subjected to PELD surgery were included. Additionally, this study is not multi-centre. All of these factors may lead to biases. However, the goal of this study was to locate the specific level that was responsible for gluteal pain in LDH. In this regard, we found that L4/5 disc hernias were a main factor in LDH-related gluteal pain. Further anatomical studies may be undertaken to support these results.

## Additional file

Additional file 1: LDH_GP.sav. (SAV 31 kb)

## Abbreviations

GP: Gluteal Pain
LBP: Low Back Pain
LDH: Lumbar Disc Herniation
PELD: Percutaneous Endoscopic Lumbar Discectomy
SCNEN: Superior Cluneal Nerve Entrapment Neuropathy
